# Cholera in Lower Animals
*Suggestions for Observations on the Influence of Cholera and other Epidemic Poisons on the Lower Animals. By W. Lauder Lindsay, M.D., Perth. Pamphlet. pp. 15. Reprinted from the *Edinburgh Medical Journal*, July 1857.


**Published:** 1857-10

**Authors:** 


					CHOLEEA IN LOWER ANIMALS *
Dr. Lauder Lindsay has for some time taken much interest
in the investigation of the question of the communicability of
cholera to the lower animals. His paper contains a series of
carefully proposed etiological and pathological problems, in
regard to which he invites contributions from observers. We
extract the chief starting questions with regard to causation :
"I. Are sporadic and epizootic diseases of the lower animals,
which have been observed prior to, during, or immediately subse-
quent to, epidemic cholera in man, not equally common at other
periods, when there is no presumptive evidence of any cholera
poison existing in the atmosphere ? If so, wherein do they differ
from, or resemble, the same diseases occurring during the preva-
lence of cholera in man ?
"II. What is the precise connection between coincident epi-
demics in plants, the lower animals, and man, in regard to causa-
tion ? Is it merely coincidental, or are they really due to a common
cause ? And, if so, what are the laws which regulate the influence
of the same atmospheric poison on these different classes of organ-
ized beings ?"
We hope Dr. Lindsay will meet with success in his endea-
vours to investigate the connexion of epidemic and epizootic
diseases.
* Suggestions for Observations on the Influence of Cholera and other
Epidemic Poisons on the Lower Animals. By W. Lauder Lindsay, M.D.,
Perth. Pamphlet, pp. 15. Reprinted from the Edinburgh Medical
Journal, July 1857.

				

